# Successful surgical epicardial cryoablation of refractory atrial tachycardia in a patient with repaired tetralogy of Fallot after multiple failed endocardial ablations

**DOI:** 10.1016/j.hrcr.2022.12.008

**Published:** 2022-12-13

**Authors:** Qasim J. Naeemah, Miyako Igarashi, Muneaki Matsubara, Tomoko Ishizu, Akihiko Nogami, Masaki Ieda

**Affiliations:** ∗ Department of Cardiology, Faculty of Medicine, University of Tsukuba, Tsukuba, Japan; † Department of Cardiovascular Surgery, Faculty of Medicine, University of Tsukuba, Tsukuba, Japan

**Keywords:** Tetralogy of Fallot, Atrial tachycardia, Catheter ablation, Surgical cryoablation, Epicardial AT focus

## Introduction

Long-term survival after tetralogy of Fallot (TOF) repair is promising, close to 95% at 25 years post–reparative surgery.^1^ Nonetheless, the prevalence of atrial tachyarrhythmia increases significantly after about 10–15 years after surgical repair.^2^ Most of these arrhythmias arise from the right atrium (RA), and cavotricuspid isthmus (CTI)-dependent atrial flutter (AFL) is the commonest identified atrial tachycardia (AT).^3^ Radiofrequency catheter ablation is successful in treating most of these arrhythmias.^3^ However, endocardial ablation is insufficient in some patients, and different therapeutic modalities are necessary. Herein, we present a case of successful surgical epicardial cryoablation of AT in a patient with repaired TOF done during pulmonary valve replacement, which was refractory to multiple endocardial catheter ablations.

## Case report

**Figure 1:**
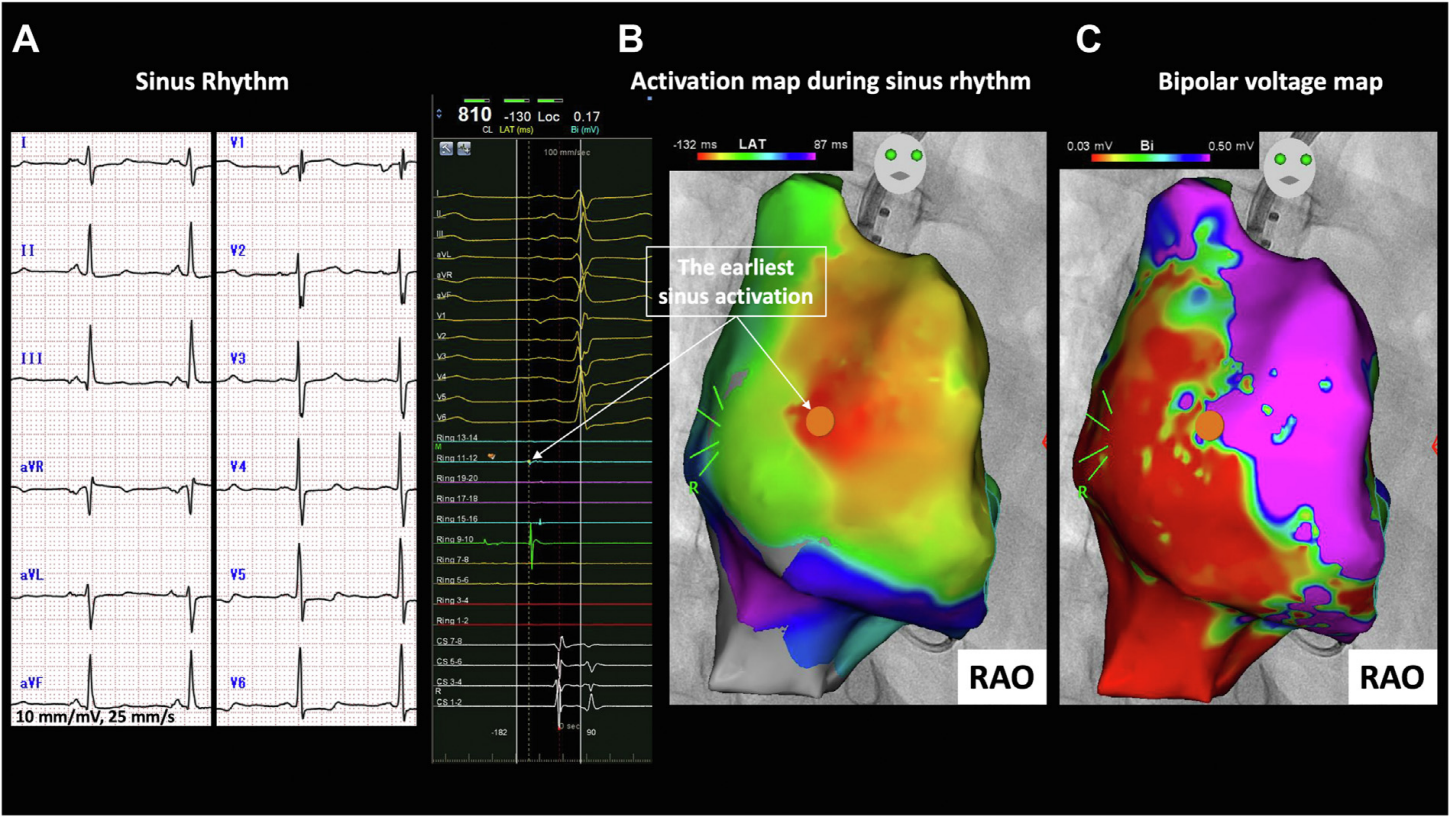
Twelve-lead electrocardiogram (ECG) and activation mapping during sinus rhythm. **A:** Incomplete right bundle branch block of 90 ms duration was evident on ECG with right axis deviation. **B:** Activation mapping of the right atrium (RA) during sinus rhythm to accurately identify the earliest sinus activation to avoid during radiofrequency energy application. **C:** The bipolar voltage map showed an extensive low-voltage area involving the RA posterolateral wall. RAO = right anterior oblique.

A 35-year-old female patient had TOF with pulmonary atresia and presented with palpitation and exercise intolerance over the last 5 years. At the age of 2 months, she underwent palliative surgery. When she was 5 years old, she underwent repair surgery with the Rastelli procedure (closing the ventricular septal defect, mono-cusp transannular patch, and placement of conduit from the right ventricle to pulmonary artery). The patient progressed well and uneventfully with regular follow-up visits. At the age of 24 years, she underwent balloon dilatation of the pulmonary artery (the conduit). The 12-lead electrocardiogram (ECG) during sinus rhythm showed incomplete right bundle branch block (QRS duration, 90 ms), PR interval of 150 ms, and right axis deviation (Figure 1A). The transthoracic echocardiography prior to the last surgery revealed a left ventricular ejection fraction of 60%, enlarged RA (RA area, 34 cm^2^), and dilated right ventricle with severe pulmonary regurgitation and moderate stenosis.

At age 30, she started to have palpitation episodes, and AT was documented by ECG. At that time, she underwent catheter ablation, and CTI-dependent AFL was ablated successfully (Supplemental Figures 1 and 2). The vascular access was via the right subclavian and jugular veins, as the patient had bilateral femoral vein occlusion. However, the palpitation episodes relapsed. The second procedure was performed in which a focal AT was targeted at the posterolateral RA wall (Supplemental Figure 3). In both sessions, atrial pacing and intravenous isoproterenol infusion could not induce another AT at the end of the procedure.

**Figure 2:**
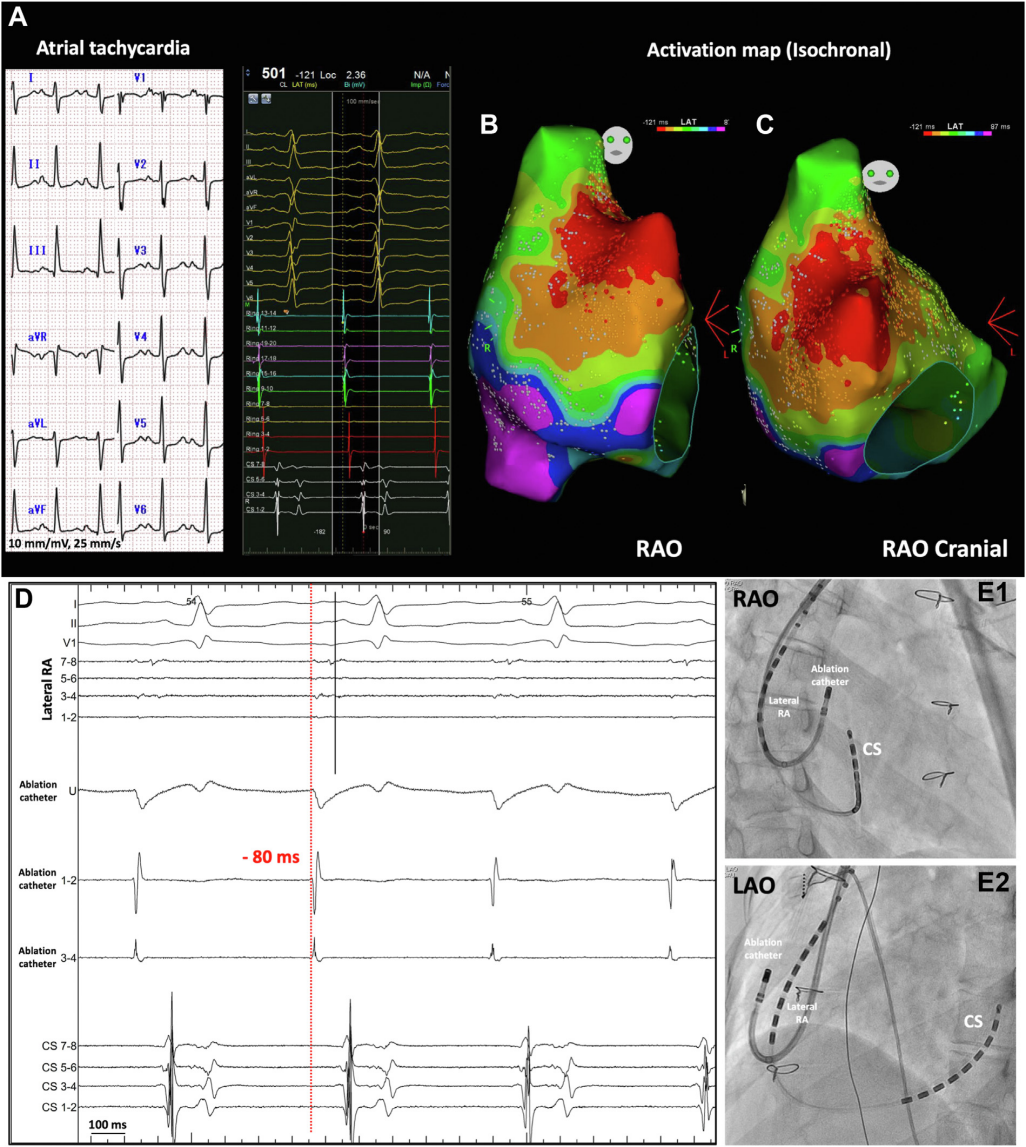
Twelve-lead electrocardiogram (ECG) and activation mapping during atrial tachycardia and the ablation site. **A:** Surface ECG showed atrial tachycardia at a rate of 120 per minute with 1:1 AV conduction. The P wave was positive in inferior leads and V_1_. **B, C:** The isochronal activation mapping of atrial tachycardia (8 equal isochrones) showed a wide area of focal activity around the right atrium (RA) appendage. This may indicate a relatively distal activation site, such as epicardial emanating focus breaking out endocardially, giving a wide activation area. **D:** The local electrogram on the distal ablation catheter (1-2) preceded the surface P wave by about 80 ms; however, the tachycardia was not perturbed despite multiple radiofrequency energy applications. **E:** Panels E1 and E2 show the location of the ablation catheter in the right anterior oblique (RAO) and left anterior oblique (LAO) views, respectively. CS = coronary sinus.

The patient had documented AT recurrence after about 1 month from the second session. In the 12-lead ECG, the P wave was positive in inferior leads and V_1_ with 1:1 AV conduction (Figure 2A). The third procedure was decided. In this session, the electroanatomic mapping system was the Carto system (Biosense Webster, Diamond Bar, CA). A duodecapolar catheter (BeeAT; Japan Lifeline, Tokyo, Japan) was inserted into the coronary sinus via the right jugular vein. A multi-spline mapping catheter (PENTARAY; Biosense Webster) was advanced into the RA through a steerable sheath (AGILIS NXT; Abbott, Abbott Park, IL) via the left subclavian vein.

At first, activation and voltage maps were created in sinus rhythm to localize the earliest atrial activation site (Figure 1B). The bipolar voltage map showed an extensive low-voltage area involving RA, relatively sparing the anterior and the septal wall (Figure 1C). Under intravenous isoproterenol infusion, AT of cycle length 500 ms was induced. This AT was mapped and revealed a focal pattern of activation. Sinus node reentry was excluded as a mechanism because of the different P-wave morphology between the sinus rhythm and AT. Also, the mode of induction with isoproterenol infusion and not atrial pacing is more compatible with abnormal automaticity. Pacing maneuvers, to further ascertain the mechanism, were not done owing to the nonsustainability of the AT with spontaneous and frequent induction and termination. The focal activity of this AT was rather unusual. It involved a wide area around the RA appendage, as evident by the isochronal activation map (Figure 2B and 2C), compared with the previous session (Supplemental Figure 3C). This may indicate a distant source of the focus relative to the mapped surface. The mapping catheter was replaced by the ablation catheter (THERMOCOOL SMARTTOUCH SF; Biosense Webster). The targeted site was 80 ms preceding the P wave (Figure 2D). This site was ablated with 30–35 W with an ablation index of about 500 and a contact force of 10–15 grams. However, multiple radiofrequency energy applications (35 applications for a total of 17 minutes) could not perturb the AT. A possibility of epicardial origin was suspected. As the patients had previous cardiac surgeries, pericardial adhesion was anticipated, and percutaneous epicardial access was not attempted. Therefore, further ablation was deemed unreasonable.

**Figure 3:**
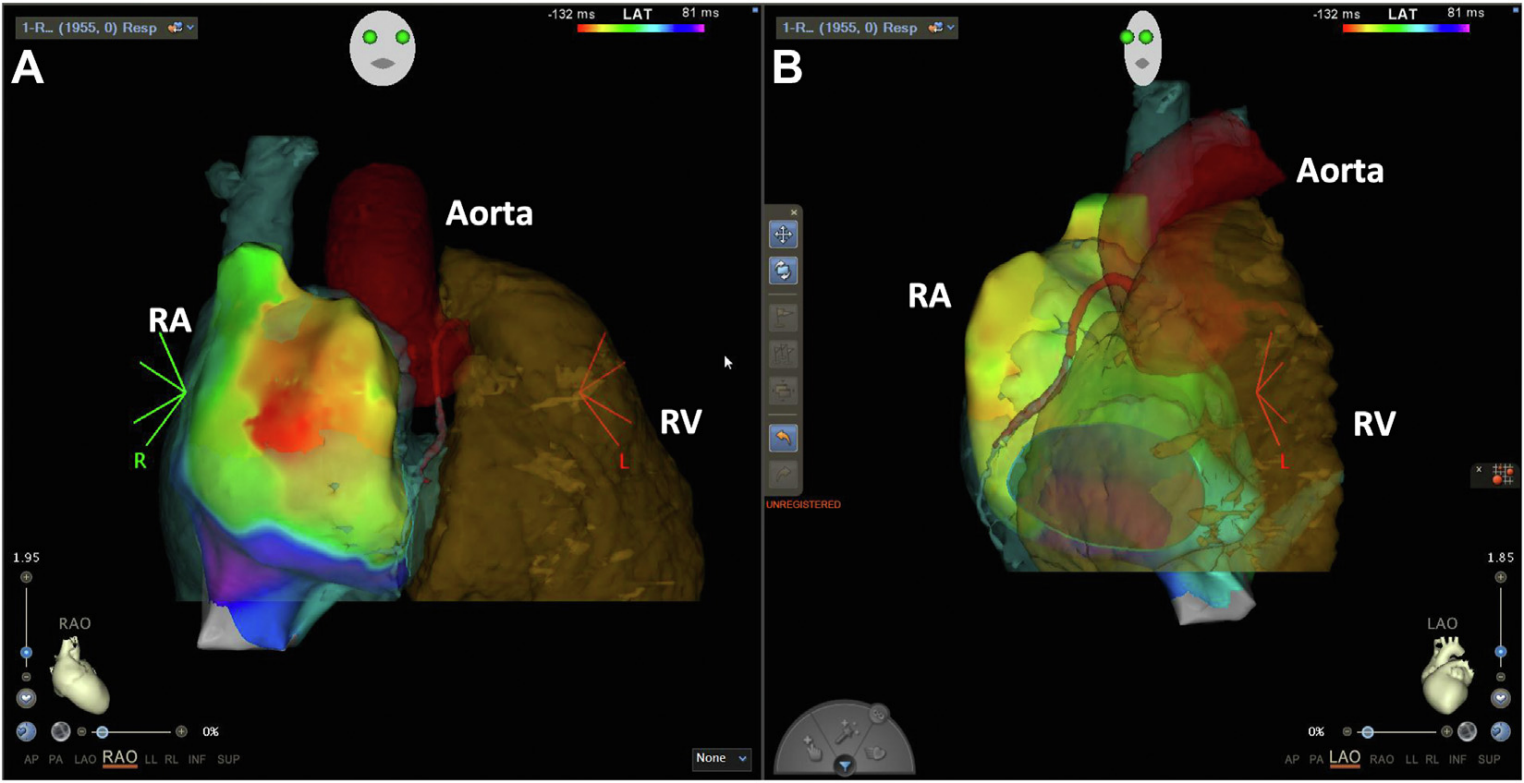
Activation map of sinus rhythm merged with cardiac computed tomography (CT) scan. **A** and **B** were the right anterior oblique (RAO) and left anterior oblique (LAO) views, respectively, showing the earliest sinus activation merged with preprocedural contrast CT scan of the heart. RA = right atrium; RV = right ventricle.

During the preceding years, the pulmonary regurgitation worsened from moderate to severe with right ventricular dilatation. The exercise capacity declined progressively. Thus pulmonary valve replacement was considered to tackle the worsening of cardiac function. During the preoperative planning, we provided the cardiac surgeon with the electroanatomic map of the RA merged with the preprocedural cardiac computed tomography scan and the site of the sinus node activation (Figure 3) and AT activation map (Figure 2B and 2C) for surgical epicardial cryoablation. In the surgical procedure, pulmonary valve replacement, reconstruction of the transannular patch, and surgical cryoablation of the RA epicardium on the presumable site of AT focus were done. The surgical cryoablation (−60°C for 90 seconds, 5 applications) with a T-shaped cryoprobe (20 mm in length and 5 mm in width; Tonokura, Tokyo, Japan) was performed during sinus rhythm with reference to the AT activation map (Figure 2B and 2C).

An intraoperative electrophysiological study was not performed because we expected difficulty in AT induction owing to the general anesthesia. In the last session, it was induced only in an awake state with intravenous isoproterenol infusion, and also it was difficult to set up the mapping system. The sinus node function was not affected. Six months have passed since the surgery, and the patient has been free of AT recurrence. The transthoracic echocardiography 3 months post-surgery showed a marked decrease in the size of the RA (23 cm^2^).

## Discussion

Catheter ablation is a feasible therapeutic strategy for treating ATs in patients with repaired TOF with satisfactory long-term outcome.^3^ The mechanism of AT in those populations was CTI-dependent AFL, intra-atrial reentry revolving around a surgical scar from the previous surgeries, or focal type, the least common mechanism identified in those patients.^3^ In this patient, the first ablation was done for CTI-dependent AFL. In the subsequent procedures, the mechanism of AT was focal according to the activation mapping of the targeted ATs. In the third ablative session, the focal activity involved a large area around the RA appendage, as shown by the isochronal map. Endocardial ablation of the earliest site could not terminate or slow down AT.

Three possibilities could explain the failure of the endocardial approach to target this AT. The first one is vascular access and catheter maneuverability. Our patient had bilateral femoral vein occlusion, which precluded accessing the heart via the inferior vena cava and necessitated a different approach through the jugular and subclavian veins to reach the RA. Thus, stability and good contact of the ablation catheter on the targeted region became an issue. We used an ablation catheter with contact force sensing capability. This could help by providing real-time information on the lesion quality. It was previously reported that a contact-sensitive catheter could successfully ablate CTI-dependent AFL in a patient with bilateral femoral vein occlusion, as in our patient.^4^ However, targeting the CTI from the above approach is not the same as targeting the RA appendage region, which may further complicate the ablative process. The anatomical barrier should also not be ignored. The structure of RA is complex, with pectinate muscles extending to the RA appendage, which may prevent the ablation catheter from contacting the true endocardial surface.^5^ This factor could be implicated as a cause of failure to suppress the AT even though the RA wall thickness was only 2.3 mm as measured by the cardiac magnetic resonance imaging and adequate radiofrequency energy applications.

The use of cryoablation could mitigate the stability issue associated with the use of radiofrequency catheter ablation. As previously demonstrated, the adhesion between the tip of the ablation catheter and the underlying tissue mediated by freezing could create a deeper lesion that reaches the AT focus.^6^ Alternatively, using half-normal saline for irrigation would also create a larger lesion, as demonstrated objectively in the preclinical studies.^7^ Moreover, it was used safely in the ablation of atrial fibrillation.^8^

The third reason is that the focal AT originated from the epicardial surface, making endocardial ablation ineffective despite good contact. In the third session, the focus of AT involved a wide area in the vicinity of the RA appendage. This raises the possibility that the focus was on the epicardial surface, giving a wide activation area on the endocardial mapping. This possibility is also supported by the effectiveness of epicardial cryoablation, as the patient had no recurrence for 6 months after surgery. To the best of our knowledge, this is the first reported case of epicardial surgical cryoablation of presumable epicardial focal mechanism in a patient with repaired TOF.

Heart failure is prevalent in congenital heart disease, including TOF, especially with advancing age. The progressive worsening of the regurgitant valves, such as pulmonary regurgitation and chamber dilatation, further exacerbates the adverse hemodynamic disturbance. These changes beget atrial arrhythmias. Arrhythmias, in return, can provoke heart failure, and the vicious cycle continues.^9^ As a part of holistic arrhythmia management, addressing structural abnormalities such as pulmonary regurgitation is required for satisfactory arrhythmia control. However, a recent study failed to show the synergistic effect of pulmonary valve replacement on arrhythmia control, emphasizing early intervention before the irreversible changes were ensured.^10^ In our case, restoring sinus rhythm and pulmonary valve replacement resulted in reverse RA remodeling.

Historically, surgery was the only curative option that was available for the treatment of several cardiac arrhythmias. With the advent of controllable radiofrequency power sources and the technological advancement of ablative catheter manufacturing, arrhythmic surgical procedures lost popularity. Traditionally cut-and-sew techniques are used to create block lines, such as the Cox-maze procedure. However, nowadays, cryoprobe is frequently used to serve the same purpose with a better safety profile.^11^

## Conclusion

Surgical cryoablation could be a viable option to treat AT in a patient with previous multiple failed endocardial ablations, further complicated by vascular access problems such as bilateral femoral vein occlusion.

**Key Teaching Points**

• A wide area of focal activity combined with an unperturbed response to radiofrequency energy application might hint at an epicardial emanating source.
• Despite the normal thickness of the right atrium (2.3 mm in our case), endocardial ablation could not suppress a possible epicardial focal atrial tachycardia (AT) owing to the anatomical complexity of the right atrial structure.
• Surgical cryoablation could be an option to manage AT refractory to medications and endocardial ablations.

## Appendix Supplementary data

Supplemental Figure 1 **12-Lead ECG of the clinical and induced atrial tachycardia with activation map.** (**A**) The clinical atrial tachycardia (AT) was evident in 12-lead ECG with positive P in all leads except aVR. **(B)** The AT that was induced and treated during the procedure; the P was negative in inferior leads, unlike the clinical AT. **(C)** The intracardiac recording of the AT. The cycle length was 260 ms. **(D)** The activation map of the AT in the right atrium. The whole cycle length was covered, and the circuit was revolving counterclockwise around the tricuspid annulus.

Supplemental Figure 2 **Entrainment pacing and activation mapping showing the block line after ablation.** (**A**) and **(B)** The entrainment pacing from 6 and 12 o’clock around the tricuspid annulus, respectively. The pacing was 20 ms faster than the AT cycle length, and the post pacing interval (PPI) was 6 ms longer than the cycle length. This confirmed the diagnosis of cavotricuspid isthmus (CTI) dependent atrial flutter. **(C)** The activation map of the right atrium under proximal CS pacing in both right anterior oblique (RAO) and RAO caudal after CTI ablation. A clear block line was evident at around 7 o’clock of the tricuspid annulus. Spit potentials were also seen across the line of the block.

Supplemental Figure 3 **12-Lead ECG and intracardiac recording of atrial tachycardia with the activation map. (A)** The 12-lead ECG of the AT with a cycle length of 460 ms. The P was positive in inferior leads and negative in V1. This AT could not be induced by atrial pacing and only induced after isoproterenol infusion. **(B)** The intracardiac recording during the AT. **(C)** The isochronal activation mapping of AT (eight equal isochrones). Unlike in the third session, the earliest activation region was smaller. **(D)** The bipolar voltage map of the right atrium during AT. The AT focus emerged from a small island of low voltage within a very low voltage region. EGM = electrogram.
