# Supplementary figures and images for: Successful surgical epicardial cryoablation of refractory atrial tachycardia in a patient with repaired tetralogy of Fallot after multiple failed endocardial ablations
Source: HeartRhythm Case Rep. 2022 Dec 13;9(3):190–4. doi: 10.1016/j.hrcr.2022.12.008 (PMC10030303; doi:10.1016/j.hrcr.2022.12.008)

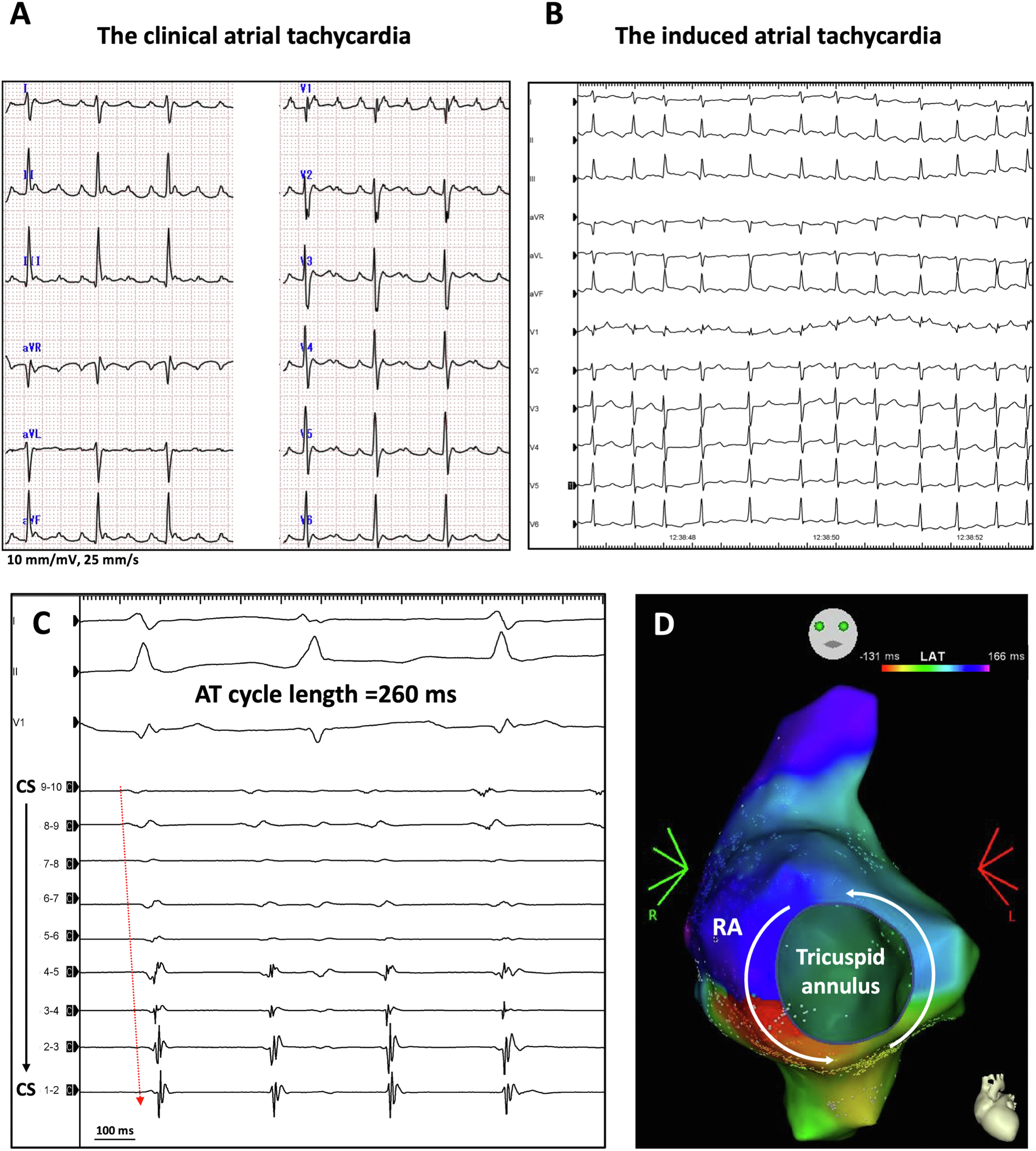

Supplement: Supplemental Figure 1 — 12-Lead ECG of the clinical and induced atrial tachycardia with activation map. (A) The clinical atrial tachycardia (AT) was evident in 12-lead ECG with positive P in all leads except aVR. (B) The AT that was induced and treated during the procedure; the P was negative in inferior leads, unlike the clinical AT. (C) The intracardiac recording of the AT. The cycle length was 260 ms. (D) The activation map of the AT in the right atrium. The whole cycle length was covered, and the circuit was revolving counterclockwise around the tricuspid annulus. [file figs1.jpg]

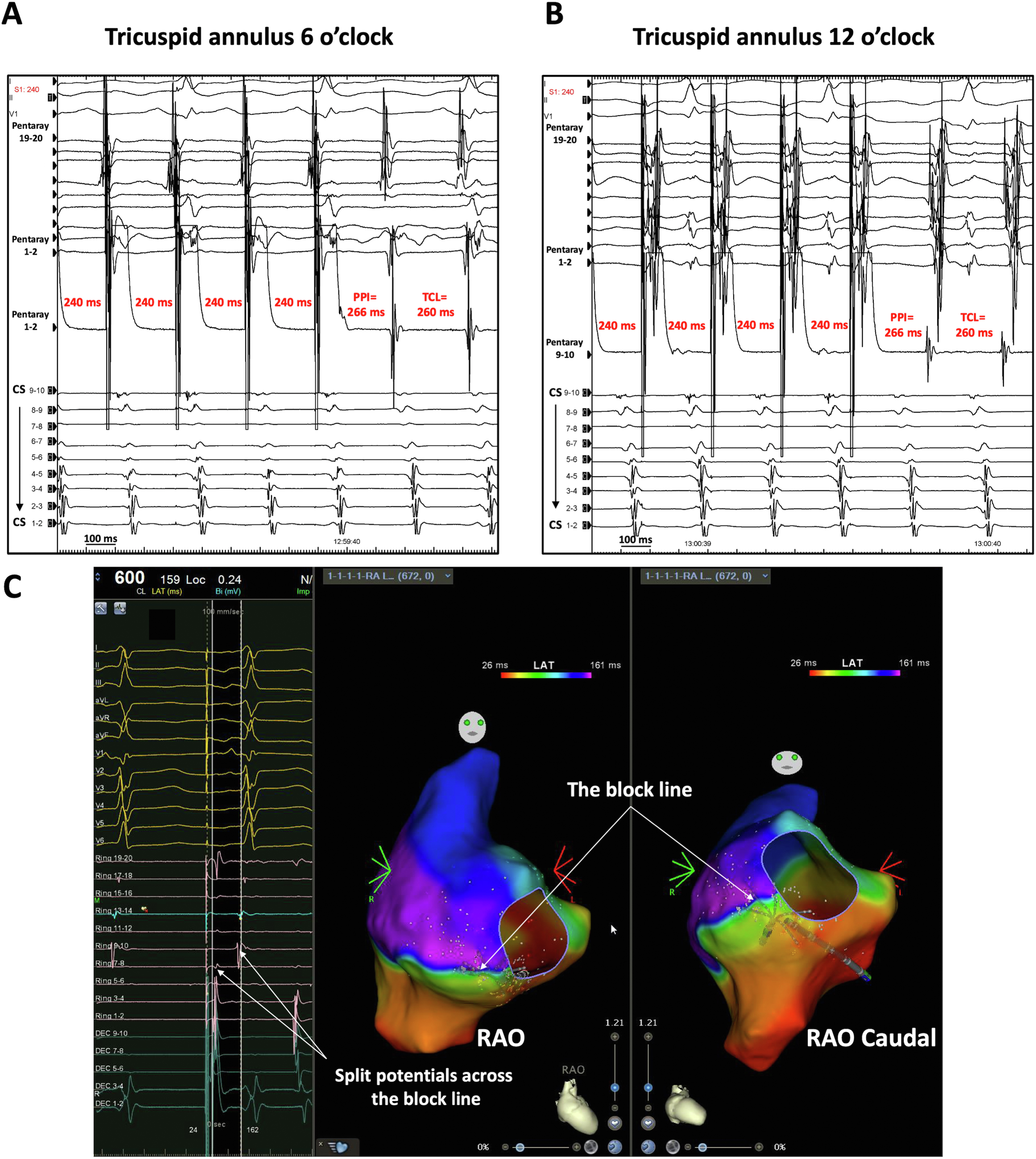

Supplement: Supplemental Figure 2 — Entrainment pacing and activation mapping showing the block line after ablation. (A) and (B) The entrainment pacing from 6 and 12 o’clock around the tricuspid annulus, respectively. The pacing was 20 ms faster than the AT cycle length, and the post pacing interval (PPI) was 6 ms longer than the cycle length. This confirmed the diagnosis of cavotricuspid isthmus (CTI) dependent atrial flutter. (C) The activation map of the right atrium under proximal CS pacing in both right anterior oblique (RAO) and RAO caudal after CTI ablation. A clear block line was evident at around 7 o’clock of the tricuspid annulus. Spit potentials were also seen across the line of the block. [file figs2.jpg]

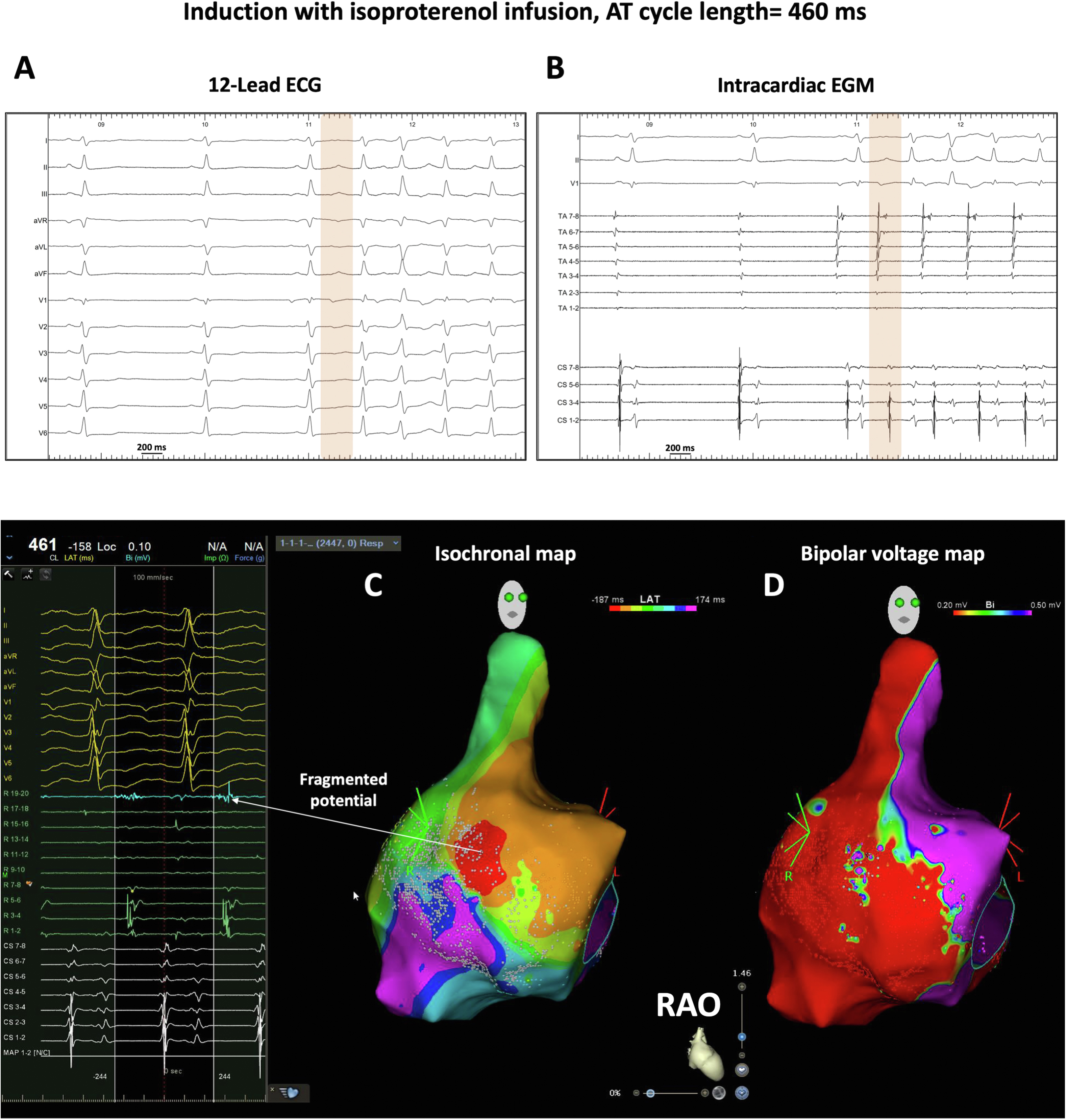

Supplement: Supplemental Figure 3 — 12-Lead ECG and intracardiac recording of atrial tachycardia with the activation map. (A) The 12-lead ECG of the AT with a cycle length of 460 ms. The P was positive in inferior leads and negative in V1. This AT could not be induced by atrial pacing and only induced after isoproterenol infusion. (B) The intracardiac recording during the AT. (C) The isochronal activation mapping of AT (eight equal isochrones). Unlike in the third session, the earliest activation region was smaller. (D) The bipolar voltage map of the right atrium during AT. The AT focus emerged from a small island of low voltage within a very low voltage region. EGM = electrogram. [file figs3.jpg]
